# The Complex Relationship of Realspace Events and Messages in Cyberspace: Case Study of Influenza and Pertussis Using Tweets

**DOI:** 10.2196/jmir.2705

**Published:** 2013-10-26

**Authors:** Anna C Nagel, Ming-Hsiang Tsou, Brian H Spitzberg, Li An, J Mark Gawron, Dipak K Gupta, Jiue-An Yang, Su Han, K Michael Peddecord, Suzanne Lindsay, Mark H Sawyer

**Affiliations:** ^1^Graduate School of Public HealthSan Diego State UniversitySan Diego, CAUnited States; ^2^Department of GeographySan Diego State UniversitySan Diego, CAUnited States; ^3^School of CommunicationSan Diego State UniversitySan Diego, CAUnited States; ^4^Department of LinguisticsSan Diego State UniversitySan Diego, CAUnited States; ^5^Department of Political ScienceSan Diego State UniversitySan Diego, CAUnited States; ^6^Division of Pediatrics Infectious DiseasesUniversity of California San Diego School of MedicineLa Jolla, CAUnited States; ^7^Epidemiology and Immunization Services BranchCity of San Diego Health and Human Services AgencySan Diego, CAUnited States

**Keywords:** Twitter, infoveillance, infodemiology, cyberspace, syndromic surveillance, influenza, pertussis, whooping cough

## Abstract

**Background:**

Surveillance plays a vital role in disease detection, but traditional methods of collecting patient data, reporting to health officials, and compiling reports are costly and time consuming. In recent years, syndromic surveillance tools have expanded and researchers are able to exploit the vast amount of data available in real time on the Internet at minimal cost. Many data sources for infoveillance exist, but this study focuses on status updates (tweets) from the Twitter microblogging website.

**Objective:**

The aim of this study was to explore the interaction between cyberspace message activity, measured by keyword-specific tweets, and real world occurrences of influenza and pertussis. Tweets were aggregated by week and compared to weekly influenza-like illness (ILI) and weekly pertussis incidence. The potential effect of tweet type was analyzed by categorizing tweets into 4 categories: nonretweets, retweets, tweets with a URL Web address, and tweets without a URL Web address.

**Methods:**

Tweets were collected within a 17-mile radius of 11 US cities chosen on the basis of population size and the availability of disease data. Influenza analysis involved all 11 cities. Pertussis analysis was based on the 2 cities nearest to the Washington State pertussis outbreak (Seattle, WA and Portland, OR). Tweet collection resulted in 161,821 flu, 6174 influenza, 160 pertussis, and 1167 whooping cough tweets. The correlation coefficients between tweets or subgroups of tweets and disease occurrence were calculated and trends were presented graphically.

**Results:**

Correlations between weekly aggregated tweets and disease occurrence varied greatly, but were relatively strong in some areas. In general, correlation coefficients were stronger in the flu analysis compared to the pertussis analysis. Within each analysis, flu tweets were more strongly correlated with ILI rates than influenza tweets, and whooping cough tweets correlated more strongly with pertussis incidence than pertussis tweets. Nonretweets correlated more with disease occurrence than retweets, and tweets without a URL Web address correlated better with actual incidence than those with a URL Web address primarily for the flu tweets.

**Conclusions:**

This study demonstrates that not only does keyword choice play an important role in how well tweets correlate with disease occurrence, but that the subgroup of tweets used for analysis is also important. This exploratory work shows potential in the use of tweets for infoveillance, but continued efforts are needed to further refine research methods in this field.

## Introduction

### Background

Use of the Internet has shifted from being solely a one-way transfer of information to an interactive multidimensional channel. Cyberspace resides as a source of information accessible to the user who is able to contribute to cyberspace through social media and online communities [1]. Infodemiology is the study of the distribution and causal factors of information in cyberspace and its ability to improve public health [2]. The Internet provides many resources for infodemiology, including search engine queries (ie, Google Flu Trends [3]), publications, marketing campaigns, and user-generated content, such as blogs and social media status updates [2]. Researchers are pioneering a variety of methods and applications using these resources for disease detection (see [4] for overview). This study focuses on the infodemiology of pertussis-related (also called whooping cough) and influenza-related status updates on Twitter (tweets).

Every year millions of Americans become infected with the flu, resulting in illness, missed work and school days, and death. Deaths from seasonal influenza occur mostly in young children and the elderly, largely because of flu complications and the exacerbation of existing conditions, such as congestive heart failure [5]. Influenza causes a substantial economic burden associated with loss in productivity because of missed work and health care costs [6]. Pertussis infects a much smaller population, but can result in severe complications, especially among those who are young and unvaccinated. Approximately 57% of infants under 1 year of age are hospitalized for pertussis, and the risk is greater the younger the child [7]. The most common complication among hospitalized infants is apnea (67%), or pauses in breathing that may result in cyanosis, followed by pneumonia (23%). Death and violent convulsions occur in approximately 1.6% of hospitalized infants, and brain disease (encephalopathy) occurs in approximately 0.4% [7]. As of December 29, 2012, Washington State had experienced 4744 pertussis cases, 5.9 times more than the prevalence for the same time period in 2011 (807) [8]. The early notification of disease outbreaks greatly increases the ability of affected communities to control and treat an epidemic. Traditional surveillance methods are a vital factor in the control of diseases, but there is often a time lag between the reporting of individual cases and the accumulation of these data into a report [9].

### Related Work

The Internet has become a fundamental tool for geographic information system (GIS) technology. Devices enabled with Global Positioning System (GPS) receivers and the Internet allow for precise geographic information of events for a variety of uses, including those focused on public health. For example, Love Clean Streets is used to alert authorities of problems in the community, such as graffiti and potholes [10]. HealthMap maps disease occurrences based on a variety of sources, including user reports [11]. Noise pollution can be analyzed based on pedestrian audio recordings from their GPS-enabled devices [12]. Researchers have used information contained in tweets to detect earthquakes in Japan [13]. Each Twitter user was labeled as a sensor; the sensor was either positive (the user tweeted earthquake-related information) or negative (they did not tweet information). Through these methods, the authors were able to detect an earthquake with 96% probability and notify authorities before the Japan Meteorological Agency [13]. Another study aimed to analyze Twitter activity during a fire outside of Marseille, France, in 2009. The researchers found tweets were accurate and timely, but not for all phases of the event. They concluded more work needed to be done in this field to fully utilize its potential [14].

Recently, other innovative syndromic surveillance methods using the Internet have been developed [15-25]. Syndromic surveillance plays a crucial role in early disease detection. In its simplest form, syndromic surveillance aims to detect a signal indicating a possible disease outbreak before the traditional surveillance methods of diagnosing and reporting diseases. The signal is usually either a symptom or symptom surrogate [26], such as pharmaceutical prescriptions [17]. Researchers in Japan found a high correlation between prescription drug purchases from over 5000 pharmacies and influenza activity reported by official sentinel surveillance [17]. Infoveillance, a component of infodemiology, is the monitoring of online texts. Online information acts as a signal of disease occurrence or public interest related to a disease [2]. These methods can be extended to investigate the public’s understanding of health topics, such as misunderstandings of dosing instructions and the resulting misuse of antibiotics [25].

The availability of public health-related Internet data has inspired many innovative studies. One study assessed the usefulness of social media for the surveillance of intentional and unintentional foodborne-illness outbreaks [9]. The authors concluded social media can play an important role in identifying clusters of foodborne illness faster than traditional methods. Many people with foodborne illness do not seek medical attention; however, they may be more likely to report symptoms online because of its ease and convenience [9]. Although limitations exist, the authors concluded that a system to exploit the large amount of data available on social media platforms in real time would be useful for detecting foodborne-illness outbreaks [9].

Twitter is the leading service of choice for disease tracking in social media. One notable study used tweets to track public concern and flu activity in the United States during the 2009 influenza A (H1N1) pandemic [22]. The researchers used tweets containing disease transmission, disease countermeasure, pork consumption, and vaccine-related keywords to track public concern. In several cases, the percentage of tweets with these keywords changed in response to news events and official disease reports [22]. A second subset of keywords was used to train a prediction model. Estimates from this model were compared with regional influenza-like illness (ILI) cases reported by the Centers for Disease Control and Prevention (CDC) and showed a close correlation. The real-time estimates from this study can be determined 1 to 2 weeks before traditional surveillance methods [22]. Researchers used Twitter to assess public concern during the 2009 H1N1 outbreak [19]. One component of the study used these more than 2 million tweets to investigate the adoption of the World Health Organization’s (WHO) terminology of H1N1 compared to swine flu, the initial term used. Over the study period, the percentage of tweets using H1N1 increased from 8.8% to 40.5% [19]. The authors concluded Twitter is a valuable tool for infodemiology, which can help health professionals to realize and address the public’s concerns [19].

### Objectives

In this study, we aimed to explore the realspace health events that influence ideas and messages in cyberspace and, in turn, determine to what extent these cyberspace messages affect the real world. More specifically, we investigated how the 2012-2013 influenza season (estimated by ILI reports) and the 2012 pertussis outbreak in Washington State are reflected in cyberspace, measured by the production of keyword-specific tweets. We also examined the extent to which these tweets act as a signal of disease occurrence or public interest, and investigated how keyword choice and specific subgroups of tweets correlate with disease occurrence, and how the scale at which disease incidence data are collected (ie, city vs state level) affects correlations with tweets collected at the city level.

## Methods

### Data Collection

This paper extends previous explorations of the innovative Visualizing Information Space in Ontological Networks (VISION) framework using the 2012-2013 flu season and the 2012 pertussis outbreak as case studies. The VISION framework was developed by our research team to better understand the connection between space, time, and messages [27]. Two information-mining tools were created: one for collecting webpage information and the other for collecting tweets. The focus of this paper was devoted exclusively to tweets under the assumption that tweet activity would be more dynamically indicative of disease diffusion compared to webpage content. Twitter provides a large source of publicly available data. Twitter has more than 140 million active users producing millions of tweets (messages of 140 characters or less) on a daily basis [28]. In this study, we tap into this resource with geo-search-enabled Twitter Tools created to operate with the Twitter Search and Streaming application programming interfaces (APIs) [29]. Our tools, in combination with the Twitter APIs, return a Microsoft Excel spreadsheet of tweets associated with a keyword (in the tweet text, the user name, or the title of a linking Web page) and within a specified geographical range. Additional information is provided with each tweet, such as the user name, the time it was created, and the location. The location is based on either the user’s self-proclaimed hometown or latitude and longitude coordinates if the user was tweeting from a GPS-enabled device.

**Figure 1:**
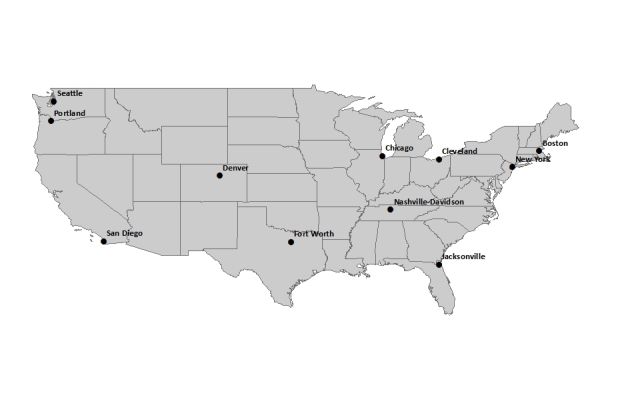
The 11 cities of interest (using a 17-mile radius) for which tweets including the keywords flu and influenza (all 11 cities) and pertussis and whooping cough (primarily Seattle and Portland) were used in the study.

In this study, tweets were collected for the keywords flu, influenza, pertussis, and whooping cough. Although the word “flu” is nested within the word influenza, our search tool treated these as separate search terms. Tweets were obtained from within a 17-mile radius of the city center of 11 US cities (Boston, MA; Chicago, IL; Cleveland, OH; Denver, CO; Fort Worth, TX; Jacksonville, FL; Nashville-Davidson, TN; New York, NY; Portland, OR; San Diego, CA; and Seattle, WA). Cities were chosen based on their population and the availability of sufficient ILI data at the city or county level. A radius of 17 miles was specified to cover a large urban area while avoiding overlapping with nearby cities. Figure 1 shows the geographical location of each of the cities of interest. For the flu and influenza keywords, tweet collection began on August 31, 2012, and continued through March 4, 2013, resulting in 161,821 flu tweets and 6174 influenza tweets. During analysis, focus was on tweets from the CDC’s Morbidity Mortality Weekly Report (MMWR) weeks 37 to 45 depending on when ILI data became available for each city (weeks starting September 1 to November 4, 2012) to MMWR week 9 (ending March 2, 2013).

The resulting tweets were compared to weekly ILI rates at the city or corresponding county level. These reports are the percentage of patients seen for influenza-like symptoms (fever greater ≥100°F and a cough and/or sore throat in the absence of a known cause other than influenza) compared to all patient visits for the week [30]. It is worth noting that ILI reporting is optional. The CDC does not report ILI data below the state level; therefore, ILI cases were obtained from individual city or county health department Web sites and, in the case of San Diego, from a contact at the County of San Diego Health and Human Services Agency [31]. In a few cases, an ILI report was missing for a particular week. In such cases, the previous and following week’s ILI percentages were averaged. In a case in which 2 weeks in a row were missing, the ILI rate for the previous week was used for the first missing week and the second missing week was derived from the following week’s ILI rate.

Tweet collection began on June 3, 2012 for the pertussis and whooping cough keywords and ended December 1, 2012, resulting in 160 pertussis tweets and 1167 whooping cough tweets. Tweets were compared to pertussis cases in Washington State. The prevalence and incidence of probable and confirmed pertussis cases in Washington State were reported on a weekly basis on the Washington State Department of Health website [8]. Tweet collection focused on 2 of the 11 cities nearest to the Washington State outbreak: Seattle and Portland.

### Analysis

The tweeting rate (number of tweets per 100,000 individuals in each city) was determined. For consistency, the city population was estimated within a 17-mile radius of the city center. The tweeting rate and disease data were then represented as bar graphs so fluctuations in each could easily be visually compared. For visualization purposes, the maximum pertussis and whooping cough tweet rates (in both Portland and Seattle) and the maximum disease incidences were rescaled and set equal to one another. For flu and influenza, the maximum tweet rate and ILI were rescaled and set equal to one another in each city.

The association between weekly aggregated tweets and disease incidence was determined using Pearson correlation coefficients in R version 2.15.1 (R Foundation for Statistical Computing, Vienna, Austria) for each of the 11 cities for influenza and Portland and Seattle for pertussis. In addition, tweets were further subdivided to determine the type of tweets that best correlated with disease cases. As mentioned previously, 4 keywords were used for tweet collection: flu, influenza, pertussis, and whooping cough. Each of these 4 datasets was segregated into nonretweets, retweets, tweets without a URL Web address, and tweets with a URL Web address. Fisher z-transformation was used to assess significant differences in correlation coefficients among various groups of tweets or keywords. Correlation coefficients were compared between tweets from the 2 keywords used for each disease, from different cities, and for the 4 tweet subgroups listed previously.

## Results

### Flu and Influenza Tweets

Correlation coefficients among flu and influenza tweet rates per 100,000 population and estimated flu incidence based on ILI reports are displayed in Table 1. Significance testing between 2 correlations was only performed when both correlations were significant individually. When comparing flu and influenza tweets from the all tweets group (column 1), correlations for both keywords were significant in 6 cities: Denver, Fort Worth, Jacksonville, Nashville-Davidson, San Diego, and Seattle. Significant differences between correlations were found in 4 of these cities (Denver, Jacksonville, San Diego, and Seattle). Among all tweets, correlations in all 4 cities were significantly higher for flu compared to influenza tweets.

Correlations differed between subgroups after subdividing the tweets for each keyword into nonretweets, retweets, tweets with a URL Web address, and those without a URL Web address. The *P* values from the Fisher z-transformations for the nonretweet versus retweet comparison and the comparison between tweets with a URL versus those without are presented in Table 1. For the flu keyword, 6 cities (Denver, Fort Worth, Jacksonville, Nashville-Davidson, San Diego, and Seattle) had significant correlation coefficients for both the nonretweet and the retweet groups. Significantly higher correlations were seen among the nonretweet group for all 6 cities (*P*<.001 for each comparison). Differences between significant nonretweet and retweet correlations for the influenza keyword were not significant. For the flu keyword, significantly larger correlations (*P*<.05 for each comparison) were found among tweets without a URL Web address compared to those with a URL Web address in 6 of the 8 cities (Boston, Cleveland, Denver, Fort Worth, Nashville-Davidson, and Seattle) in which both correlations being compared were significant. For influenza, 5 cities (Denver, Fort Worth, Nashville-Davidson, New York, and Seattle) had significant correlations for both tweets with a URL Web address and those without, but none of these comparisons showed significant differences between correlations.

**Figure 2:**
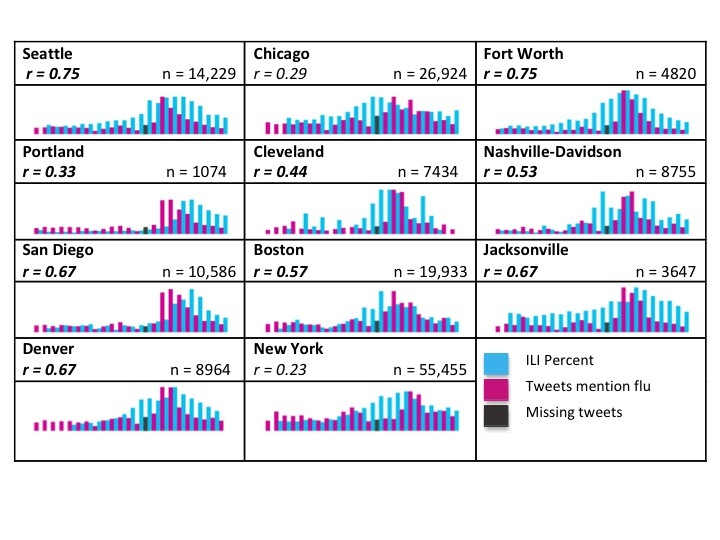
Barcharts indicating trends in all tweets containing the keyword flu (pink) and influenza-like illness (ILI) rates (blue) beginning MMWR weeks 37-45 (starting September 1 to November 4, 2012 depending on when ILI data became available for a particular city) and ending MMWR week 9 (March 2, 2013). The black bar indicates a week in which tweets were missing. Significant correlations are bolded.

**Figure 3:**
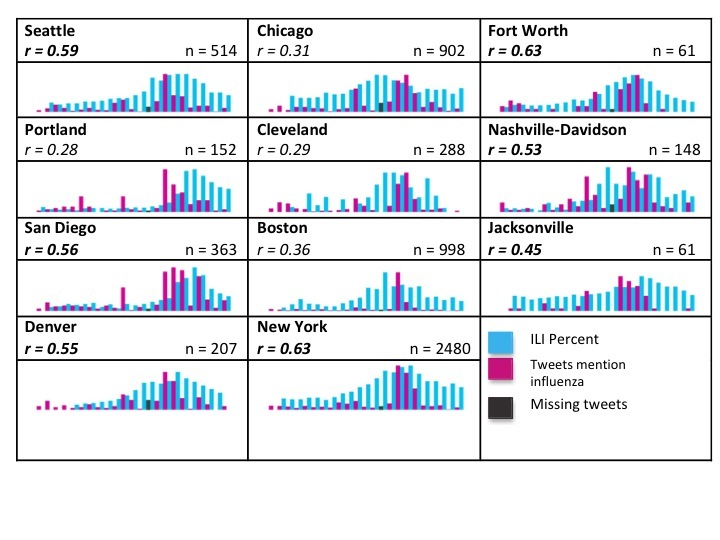
Barcharts indicating trends in all tweets containing the keyword influenza (pink) and influenza-like illness (ILI) rates beginning MMWR weeks 37-45 (starting September 1 to November 4, 2012 depending on when ILI data became available for a particular city) and ending MMWR week 9 (March 2, 2013). The black bar indicates a week in which tweets were missing. Significant correlations are shown in bold.

**Figure 4:**
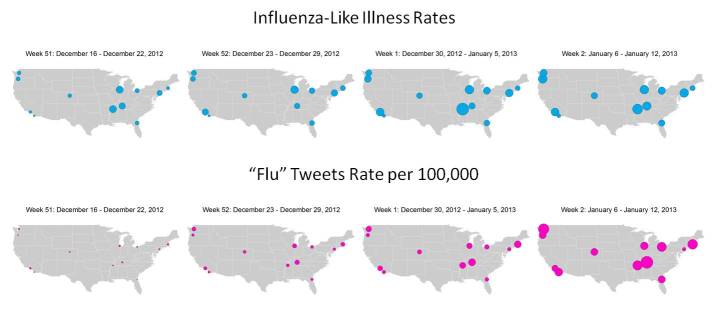
Weekly changes in influenza-like illness (ILI) rates and the rate of tweets including the keyword flu per 100,000 people starting from MMWR week 51 (December 16 to December 22, 2012) through MMWR week 2 (January 6 to January 12, 2013) mapped across the 11 cities from which tweets were collected. Larger circles represent higher rates.

The 11 cities used for tweet and ILI comparison were distributed across the continental United States, allowing for the investigation of geographical variations. Figures 2 and 3 show the weekly tweeting rate and ILI report percentages from MMWR week 39 (starting September 23, 2012) to MMWR week 9 (ending March 2, 2013) as barcharts for the flu and influenza keywords, respectively. The barcharts are organized in the table according to the corresponding city’s geographical region. The first column is more generally the western states, the second northeastern, and the third column southern states. Weekly changes in tweeting rate and ILI report percentages can be seen from MMWR week 51 (starting December 16, 2012) to MMWR week 2 (starting January 6, 2013) in Figure 4.

In Figures 2 and 3, the total (unsubdivided) tweets are shown in which the corresponding correlation coefficients were pulled from all tweets. The black bars indicate missing tweets during MMWR week 52. The black bar shows the tweets actually collected, but there were likely more. The maximum ILI and tweet rates for each city were rescaled and set equal to one another for better visualization, but this limits the viewer’s ability to compare frequency values between cities. Instead, we suggest focusing on the general trends and correlations between ILI rates and tweets within each city.

**Table 1:** Correlation coefficients between tweets (and tweet subgroups) and influenza-like illness (ILI) reports for each city for the flu and influenza keywords.

City		All tweets *r*	Nonretweet *r*	Retweets *r*	*P* value^a^	Tweets with URL *r*	Tweets without URL *r*	*P* value^b^	Total tweets n
**Flu**									
	Boston	.57	.57	.48	<.001	.49	.60	<.001	19,933
	Chicago	.29	.31	.19	<.001	.14	.40	<.001	26,924
	Cleveland	.44	.49	.30	<.001	.40	.46	.004	7434
	Denver	.67^c^	.69	.53	<.001	.62	.69	<.001	8964
	Fort Worth	.75	.75	.67	<.001	.65	.77	<.001	4820
	Jacksonville	.67^c^	.71	.32	<.001	.63	.67	.06	3647
	Nashville-Davidson	.53	.61	.35	<.001	.37	.66	<.001	8755
	New York	.23	.23	.17	<.001	.29	.17	<.001	55,455
	Portland	.33	.49	.33	<.001	.37	.52	<.001	1074
	San Diego	.67^c^	.70	.55	<.001	.66	.68	.07	10,586
	Seattle	.75^c^	.77	.67	<.001	.73	.75	.01	14,229
**Influenza**									
	Boston	.36	.41	.32	.10	.34	.46	.07	998
	Chicago	.31	.30	.27	.66	.26	.41	.02	902
	Cleveland	.29	.31	.25	.59	.35	-.06	.001	288
	Denver	.55^c^	.60	.42	.17	.49	.60	.27	207
	Fort Worth	.63	.65	.08	.24	.52	.48	.85	61
	Jacksonville	.45^c^	.45	.27	.63	.53	.28	.27	61
	Nashville-Davidson	.53	.53	.29	.35	.48	.49	.94	148
	New York	.63	.65	.61	.11	.63	.58	.07	2480
	Portland	.28	.31	.08	.35	.09	.59	.001	152
	San Diego	.56^c^	.58	.48	.30	.58	.31	.01	363
	Seattle	.59^c^	.67	.42	<.001	.55	.63	.19	514

^a^From Fisher z-transformation comparing nonretweet and retweet correlation coefficients.

^b^From Fisher z-transformation to determine significant differences among correlation coefficients of tweets with a URL compared to those without a URL Web address.

^c^Significant differences between the flu and influenza correlation coefficients for all tweets when both correlations being compared were significant.

### Pertussis and Whooping Cough Tweets

The weekly pertussis and whooping cough tweets also resulted in varying levels of correlation within cities, and by keywords and tweet subgroups (listed in Table 2). Significant correlations between tweets and pertussis incidence in Washington State were only found among tweets collected using the whooping cough keyword. This may be driven by the relatively small number of tweets for the pertussis keyword. The whooping cough keyword appeared to be more highly correlated with pertussis incidence than pertussis keyword tweets, probably reflecting the colloquial nature of tweet language. Further interpretation focuses solely on the whooping cough tweet results. Similar to the flu analysis, Fisher z-transformation was used when testing for significant differences between 2 individually significant correlations. Among the all tweets group, tweets from Portland were significantly more highly correlated with disease incidence than tweets from Seattle (*P*<.001).

Tweets were divided into nonretweets, retweets, tweets with a URL Web address, and tweets without a URL Web address, but trends between these groups were not as obvious as with the flu analysis. Correlations for nonretweets and retweets were both significant in Portland. Although nonretweets appeared to be more highly correlated with disease incidence, the difference was not significant (*P*=.39). On the other hand, correlations were significant for both tweets with a URL Web address and those without a URL Web address among tweets from Seattle. In this case, tweets without a URL Web address were significantly more highly correlated with pertussis incidence in Washington State than tweets with a URL Web address (*P*=.01).

**Figure 5:**
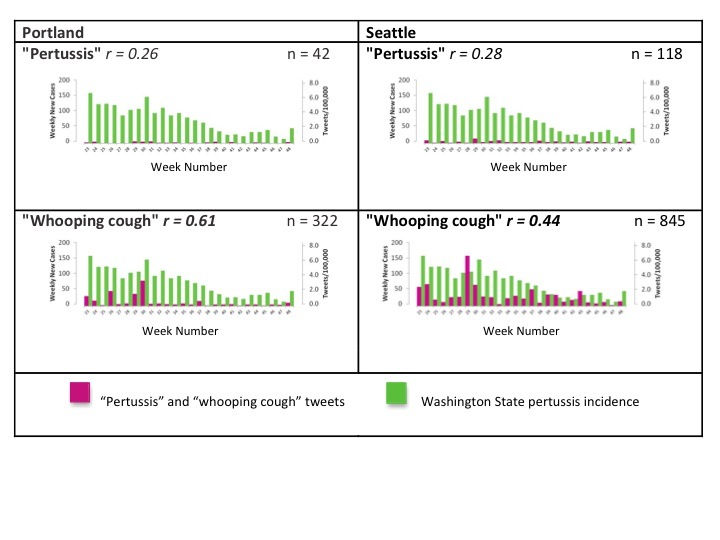
Barcharts indicating trends in all tweets containing the keywords pertussis and whooping cough (pink) and pertussis incidence in Washington State (green) for Portland and Seattle beginning MMWR weeks 23-48 (June 3, 2013 to December 1, 2013). Significant correlations are bolded.

Figure 5 gives a visual representation of all pertussis and whooping cough tweets compared to pertussis incidence from MMWR weeks 23 to 48 (June 3 and ending December 1, 2012). Significant correlations among the all tweets group and disease incidence are shown in bold. The highest tweeting rate and the largest weekly incidence were rescaled and set equal to one another for better visualization. This figure illustrates the difference in tweeting rates between the 2 cities. There were relatively few pertussis tweets (top row) compared to whooping cough tweets (bottom row). Referring back to Table 2, it is apparent that tweets from Portland were more highly correlated with pertussis incidence in Washington State than those from Seattle for the whooping cough keyword.

**Table 2:** Correlation coefficients between tweets (and tweet subgroups) and pertussis incidence in Washington State in Seattle and Portland for the pertussis and whooping cough keywords.

City		All tweets *r*	Nonretweets *r*	Retweets *r*	*P* value^a^	Tweets with URL *r*	Tweets without URL *r*	*P* value^b^	Total tweets n
**Pertussis**									
	Portland	.26	.21	.26	.90	.27	.13	.69	42
	Seattle	.28	.21	.31	.65	.25	.15	.58	118
**Whooping cough**									
	Portland	.61^c^	.56	.49	.39	.53	.38	.28	322
	Seattle	.44^c^	.47	.37	.11	.41	.44	.01	845

^a^From Fisher z-transformation comparing nonretweet and retweet correlation coefficients.

^b^From Fisher z-transformation to determine significant differences among correlation coefficients of tweets with a URL compared to those without a URL Web address.

^c^Significant differences between the Seattle and Portland correlation coefficients for all tweets.

## Discussion

### Principal Findings

This study demonstrates that tweets can function as a signal of disease activity and public interest. In this paper, we outline the differences in the ability of varying groups of tweets to temporally track influenza and pertussis incidence measured in the community. Correlations between tweets and local disease activity were variable, but were relatively strong in some areas and for specific subgroups of tweets, such as nonretweets and those without a URL Web address. Another key finding is the benefit of using the vernacular term for diseases, such as whooping cough rather than pertussis.

The 2012-2013 influenza season was unique in its temporal and spatial spread. The 2012-2013 flu season peaked earlier than it had in almost a decade [32] and cases were initially seen on the east coast. Referring to Table 1 and the charts in Figures 2 and 3, stronger correlations occurred in western cities compared to northeastern cities. Because of the higher number of tweets and the more significant correlations, our interpretation will focus on Figure 2. For the most part, both tweeting rates and ILI rates are low for the first two-thirds of the time period, then peak and then decrease in the last third. Among the 9 cities that had significant correlations between tweets and ILI rates, tweeting rates peaked before ILI rates in 5 cities. This shows tweets have the potential to act as a signal of a flu outbreak before traditional ILI reporting methods. However, this was only evident in 5 cities; in the other 4, ILI peaked before or at the same time as the tweets. Further investigation is needed to determine what combination of keywords or tweet subgroups can indicate a looming outbreak before traditional methods.

Nonretweets and those tweets without a URL Web address were more highly correlated with ILI activity compared to retweets and tweets with a URL Web address, respectively. Retweets and tweets with a URL Web address are arguably less likely to be about the individual tweeting them, and are likely used for sharing information created by others. Nonretweets and tweets without a URL Web address may have correlated better with flu activity than their respective counterparts because users are tweeting about themselves and possibly indicating that they have the flu. When Twitter users indicate they are infected with the flu, it is not possible to know if this is after a health provider’s diagnosis or, more likely, the users’ own interpretations of their symptoms.

On the other hand, the pertussis outbreak in Washington State provided a unique opportunity for analyzing the interaction between cyberspace and realspace after the disease incidence had peaked. In general, correlation coefficients between tweets and pertussis incidence were lower than those for tweets and ILI rates, but this may have been because of fewer tweets and the nature of the diseases. Figure 5 shows just how low the pertussis tweet frequencies were compared to whooping cough. We assumed tweets from Seattle would correlate better with pertussis incidence in Washington given that Seattle is in the state, but this was not the case. In fact, correlations were higher outside of Washington State (in Portland) for 4 of 5 tweet categories (all except tweets without a URL Web address).

Because of the larger tweet number and significant results, our interpretations will focus on the whooping cough keyword. Like the influenza analysis, tweets without a URL Web address were more strongly correlated with pertussis incidence than tweets with a URL Web address, but this was only significant in Seattle. Correlations among nonretweets remained higher compared to retweets, although this was not significant in either city.

Referring back to Figure 5, it can be seen that the pertussis incidence starts high and then slowly decreases over the 25 weeks. Overall, whooping cough tweets increased until week 29 and then decreased for the rest of the time period. The peak in tweets during week 29 was likely driven by media reports on a CDC press release about the pertussis epidemic in Washington State [33]. In fact, many of the tweets during that week mentioned the press release and were accompanied by a URL Web address. It seemed in this analysis the media played a larger role in tweet production than in the flu analysis. If nothing else, these findings suggest that health communication campaigns can penetrate social media in measurable ways. Because tweet collection began after the number of new pertussis cases peaked, we are unable to conclude whether tweets could signal a potential pertussis outbreak. However, because of the correlation between tweets and pertussis in Portland, for example, further explorations in this area may prove to be worthwhile.

Differences between trends seen for influenza and pertussis may have been driven by several factors. First, the 2 diseases are measured differently. Pertussis is a mandatory reported disease, meaning the Washington State pertussis incidence rates were based on true diagnosed cases. Influenza cases, on the other hand, were measured by a proxy. The ILI diagnoses can only estimate possible influenza cases, but may also be an indicator of other respiratory diseases. There were fewer pertussis-related tweets than influenza-related tweets. This may reflect the real world prevalence of the diseases. Each year, many people become infected with the flu, whereas relatively few are infected with pertussis. The commonality of flu and the rarity of pertussis make outbreaks a topic of interest in the media.

The age range of infection may also play a role in differing trends between influenza and pertussis correlations with tweets. People of all ages become infected with the flu, including a large adult population. However, pertussis is more common and most severe in infants and young children. This younger population may be less likely or unable to tweet about their illness. Also, the CDC press release resulted in a large increase in tweets, many of which contained a URL Web address linking to an article on this topic.

In addition, we were able to investigate how well tweets collected at the city level correlated with disease at the city or state level. As outlined previously, we have several conjectures as to why the flu analysis showed better correlations with disease occurrence than the pertussis analysis, but it is also important to emphasize that tweets were collected at the city level and compared to city ILI rates or state-level pertussis incidence. We chose to focus on the city level because of the importance of a quick local response in keeping the disease from spreading. Usually, ILI rates are aggregated into larger regions because of limitations in reporting at a smaller scale. However, 1 study during the 2009 H1N1 influenza season reported that ILI rates from their 8 sentinel sites that were part of a university health care system correlated well with state and regional ILI rates and were available sooner [34].

Differences in correlation coefficients may have also been driven by tweets from Seattle and Portland, which may not have been representative of Washington State pertussis activity. As a basis for contrast and control, it would have been beneficial to have either collected tweets from more cities in Washington State or to have obtained pertussis incidence at the city level for Seattle and Portland. This could also have shed light on the reasons why tweets from Portland were more highly correlated with pertussis incidence than those from Seattle. Further exploration of the geography of disease outbreak becomes an important direction for future research.

A few similarities between the influenza and pertussis analyses are also evident. For both, higher correlations were seen among nonretweets versus retweets. A hypothesis for this trend has already been given for influenza, but in the case of pertussis, an explanation is elusive at present. Both the influenza and pertussis explorations indicated the keyword chosen for collecting tweets played a vital role in correlation coefficients. As expected, there were more whooping cough than pertussis tweets, possibly because whooping cough is the colloquial term, whereas pertussis is used primarily by health professionals. A similar trend was seen between the flu and influenza tweets; both were used interchangeably by the general public, but flu may have been the preferred term among Twitter users because of the character limit of each tweet.

### Limitations

Limitations in this study were experienced for both tweet collection and disease reporting. Server issues interrupted the VISION information-mining tool and resulted in missing flu and influenza tweets during MMWR week 52 (the week is indicated by black bars in Figures 2 and 3). We suspect that if some tweets were not missing, correlations between tweets and ILI rates would have been slightly different and possibly higher. On the other end, ILI reporting is optional and the health care providers who supplied ILI rates varied between cities. For example, in some cities ILI was reported by emergency departments, whereas ILI was reported by primary care physicians in others. These 2 sources may have different ILI rates, but the general trend over time is likely similar in both. For the pertussis and whooping cough keywords, tweet collection did not begin until the number of new pertussis cases in Washington State had already peaked. Obviously, this makes it impossible to evaluate whether tweets in this case could detect an outbreak before traditional methods. However, in Portland, for example, moderate to strong correlations were observed after the outbreak, indicating an association between whooping cough tweets and pertussis. Further exploration is needed to determine whether this trend would have preceded the peak in new pertussis cases.

The number of keywords used in this study was fairly restricted because of the exploratory nature of this work. Additional keywords may greatly influence the correlations observed between tweets and disease occurrence. These additional keywords may include those in other languages, especially in cities with a large population of non-English or multilingual speakers. Another limitation is the possible misclassification of tweets by location. A previous study indicated only approximately 2.02% of the 23.8 million tweets collected worldwide during 2 separate weeks in October and November 2011 were accompanied by a GPS location [35]. For those tweets without a GPS location, we relied on the user’s self-proclaimed hometown; however, the meaning of hometown can vary. The same study investigated the accuracy of user-supplied data in the United States by comparing tweets that contained both a GPS location and a user-supplied location. The state was determined for both and found to match approximately 88% of the time [35]. Although this study focused on tweets at the city level, previous work indicates self-proclaimed hometowns may be reliable. Furthermore, tweets were collected during the flu season and during the pertussis epidemic; however, it may be beneficial to collect tweets throughout the year to better determine how well tweets can detect the initial outbreak.

### Conclusions

Because of the ever-changing nature of cyberspace, and specifically social media, the use of Internet data for infodemiology and infoveillance research provides many challenges. The meanings of messages change over time and within spatial variations leaving a complex system for researchers to navigate. However, exploratory results from this study indicate a strong association between tweets in cyberspace and the real world events of disease occurrence.

In future work, we aim to further investigate the actual tweet content and its association with disease incidence at the city, state, and country level. In addition, attention needs to be given to the impact the media have on the population’s tweeting rate; for example, Twitter users may be inspired to tweet in reaction to a particular news story. Further investigation may indicate what type of tweets or specific words within these tweets best correlate with disease activity and should be used to detect outbreaks of disease in real time. Research has shown that although infoveillance methods are still relatively new, their impact in detecting outbreaks is becoming more demonstrable. Well-developed infoveillance methods may detect disease diffusion weeks before traditional methods and at much lower cost, allowing health services to better prepare for and prevent disease. Continued efforts in this field are needed to reach the potential of infodemiology to improve the public’s health and, specifically, its application in syndromic surveillance.

## Abbreviations

API: application programming interface
CDC: Centers for Diseases Control and Prevention
GIS: geographic information system
GPS: Global Positioning System
ILI: influenza-like illness
MMWR: Morbidity Mortality Weekly Report
VISION: Visualizing Information Space in Ontological Networks
WHO: World Health Organization

## References

[1] Kaplan AM, Haenlein M (2010). Users of the world, unite! The challenges and opportunities of Social Media. Bus Horiz.

[2] Eysenbach G (2009). Infodemiology and infoveillance: framework for an emerging set of public health informatics methods to analyze search, communication and publication behavior on the Internet. J Med Internet Res.

[3] Ginsberg J, Mohebbi MH, Patel RS, Brammer L, Smolinski MS, Brilliant L (2009). Detecting influenza epidemics using search engine query data. Nature.

[4] Brownstein JS, Freifeld CC, Madoff LC (2009). Digital disease detection--harnessing the Web for public health surveillance. N Engl J Med.

[5] Centers for Disease Control and Prevention (2009). Seasonal Influenza (Flu).

[6] Molinari NA, Ortega-Sanchez IR, Messonnier ML, Thompson WW, Wortley PM, Weintraub E, Bridges CB (2007). The annual impact of seasonal influenza in the US: measuring disease burden and costs. Vaccine.

[7] Centers for Disease Control and Prevention (2012). Pertussis (Whooping cough).

[8] Washington State Department of Health.

[9] Henning K (2004). Center for Disease Control and Prevention.

[10] Love Clean Streets.

[11] HealthMap.

[12] Kanjo E (2009). NoiseSPY: a real-time mobile phone platform for urban noise monitoring and mapping. Mobile Netw Appl.

[13] Sakaki T, Okazaki M, Mastsuo Y (2010). Earthquake shakes Twitter users: Real-time event detection by social sensors.

[14] De Longueville B, Smith R, Luraschi G (2009). "OMG, from here, I can see the flames!": a use case of mining Location Based Social Networks to acquire spatio-temporal data on forest fires. Proceedings of the 2009 International Workshop on Location Based Social Networks.

[15] Arranz Izquierdo J, Leiva Rus A, Carandell Jäger E, Pujol Buades A, Méndez Castell MC, Salvà Fiol A, Esteva Cantó M (2012). [Syndromic surveillance of Influenza-like illness in primary care: a complement to the sentinel surveillance network for periods of increased incidence of Influenza]. Aten Primaria.

[16] Culotta A (2012). Lightweight methods to estimate influenza rates and alcohol sales volume from Twitter messages. Lang Resources & Evaluation.

[17] Ohkusa Y, Sugawara T, Taniguchi K, Okabe N (2011). Real-time estimation and prediction for pandemic A/H1N1(2009) in Japan. J Infect Chemother.

[18] Achrekar H, Gandhe a, Lazarus R, Yu S, Liu B (2011). Predicting flu trends using Twitter data.

[19] Chew C, Eysenbach G (2010). Pandemics in the age of Twitter: content analysis of Tweets during the 2009 H1N1 outbreak. PLoS One.

[20] Chunara R, Andrews JR, Brownstein JS (2012). Social and news media enable estimation of epidemiological patterns early in the 2010 Haitian cholera outbreak. Am J Trop Med Hyg.

[21] Heaivilin N, Gerbert B, Page JE, Gibbs JL (2011). Public health surveillance of dental pain via Twitter. J Dent Res.

[22] Signorini A, Segre AM, Polgreen PM (2011). The use of Twitter to track levels of disease activity and public concern in the U.S. during the influenza A H1N1 pandemic. PLoS One.

[23] Sofean M, Smith M (2012). A real-time disease surveillance architecture using social networks. Stud Health Technol Inform.

[24] Newkirk RW, Bender JB, Hedberg CW (2012). The potential capability of social media as a component of food safety and food terrorism surveillance systems. Foodborne Pathog Dis.

[25] Scanfeld D, Scanfeld V, Larson EL (2010). Dissemination of health information through social networks: twitter and antibiotics. Am J Infect Control.

[26] Henning K Centers for Disease Control and Prevention.

[27] Tsou MH, Yang JA, Lusher D, Han S, Spitzberg B, Gawron JM, Gupta D, An L (2012). Mapping social activities and concepts with social media (Twitter) and web search engines (Yahoo and Bing): a case study in 2012 U.S. presidential election.

[28] Twitter blog (2012). Twitter.

[29] (2012). Twitter Developers.

[30] Centers for Disease Control and Prevention (2012). Influenza (flu): Overview of influenza surveillance in the United States.

[31] (2012). SDSU Mapping Ideas.

[32] Centers for Disease Control and Prevention (2012). Press Briefing Transcript.

[33] Centers for Disease Control and Prevention (2012). Press Briefing Transcript.

[34] Baker AW, Enfield K, Mehring B, Turner JC, Sifri CD (2012). Local influenza-like illness surveillance at a university health system during the 2009 H1N1 influenza pandemic. Am J Infect Control.

[35] Burton SH, Tanner KW, Giraud-Carrier CG, West JH, Barnes MD (2012). "Right time, right place" health communication on Twitter: value and accuracy of location information. J Med Internet Res.

